# Innate Immune System Activation and Neuroinflammation in Down Syndrome and Neurodegeneration: Therapeutic Targets or Partners?

**DOI:** 10.3389/fnagi.2021.718426

**Published:** 2021-09-16

**Authors:** Md. Mahiuddin Ahmed, Noah R. Johnson, Timothy D. Boyd, Christina Coughlan, Heidi J. Chial, Huntington Potter

**Affiliations:** ^1^Department of Neurology, University of Colorado Anschutz Medical Campus, Aurora, CO, United States; ^2^University of Colorado Alzheimer’s and Cognition Center, University of Colorado Anschutz Medical Campus, Aurora, CO, United States; ^3^Linda Crnic Institute for Down Syndrome, University of Colorado Anschutz Medical Campus, Aurora, CO, United States; ^4^Partner Therapeutics, Inc., Lexington, MA, United States

**Keywords:** innate immune system, inflammation, GM-CSF (granulocyte-macrophage colony-stimulating factor), Down syndrome, Alzheimer’s disease, apolipoprotein E, drug repurposing and discovery, amyloid-β

## Abstract

Innate immune system activation and inflammation are associated with and may contribute to clinical outcomes in people with Down syndrome (DS), neurodegenerative diseases such as Alzheimer’s disease (AD), and normal aging. In addition to serving as potential diagnostic biomarkers, innate immune system activation and inflammation may play a contributing or causal role in these conditions, leading to the hypothesis that effective therapies should seek to dampen their effects. However, recent intervention studies with the innate immune system activator granulocyte-macrophage colony-stimulating factor (GM-CSF) in animal models of DS, AD, and normal aging, and in an AD clinical trial suggest that activating the innate immune system and inflammation may instead be therapeutic. We consider evidence that DS, AD, and normal aging are accompanied by innate immune system activation and inflammation and discuss whether and when during the disease process it may be therapeutically beneficial to suppress or promote such activation.

## Introduction

Down syndrome (DS), most often caused by triplication of human chromosome 21 (Hsa21), is the most common genetic cause of both intellectual disability (ID) and age-associated cognitive decline (Epstein, 1990; Chapman and Hesketh, 2000; Silverman, 2007), affecting 1 in 700–1,000 live births worldwide (Centers for Disease Control and Prevention (CDC), 2006; Irving et al., 2008; Loane et al., 2013). The amyloid precursor protein (APP) plays a major role in the pathophysiology of Alzheimer’s disease (AD), and because the *APP* gene resides on chromosome 21, its additional copy is primarily responsible for the fact that all people with DS develop AD brain pathology, including amyloid-β (Aβ) plaques and cerebral amyloid angiopathy, by age 40 (Epstein, 1990; Snyder et al., 2020). Additionally, adults with DS develop neurofibrillary tangles of hyperphosphorylated tau, oxidative stress, vascular abnormalities, and chronic neuroinflammation, which are pathologies also present in patients with AD or other neurodegenerative diseases (Wisniewski et al., 1985; Head et al., 2016; Snyder et al., 2020).

Despite substantial epidemiological, biochemical, and genetic evidence in support of the amyloid cascade hypothesis (Hardy, 2009), the AD pathogenic pathway can be modulated by other aspects of brain physiology, especially the innate immune system and neuroinflammation (Potter, 2001; El Khoury et al., 2007; Cribbs et al., 2012; Lambert et al., 2013; Bettcher et al., 2018; Barroeta-Espar et al., 2019; Taipa et al., 2019). Alois Alzheimer first suggested a potential role for inflammation in AD based on his observation of abnormal glial cells surrounding amyloid deposits (Alzheimer, 1907). The discovery that specific inflammatory proteins, such as the cytokine interleukin-1 (IL-1) and the inflammation/acute-phase protein α_1_-antichymotrypsin (ACT), were upregulated in the AD brain and were associated with amyloid deposits solidified these early clues (Abraham et al., 1988; Mrak and Griffin, 2001; McGeer et al., 2006).

Inflammation is a complex multifactorial process in both the central nervous system (CNS) and the periphery, the activity of which varies depending on the disease stage. Microglia are the primary cell type associated with the innate immune system and neuroinflammation in the brain, with growing evidence suggesting that other cells, including astrocytes, neurons, oligodendrocytes, and pericytes also play significant roles, and brain inflammation in age-associated AD differs from that in DS-associated AD (Perry and Gordon, 1988; Colton and Wilcock, 2010; Wilcock et al., 2015). Thus, neuroinflammation may play a pivotal role in the development of AD (Akiyama et al., 2000), but the underlying mechanisms driving this pathological manifestation and its association with DS remain poorly understood.

In this mini-review, we first discuss the evidence that innate immune system activation and inflammation characterize both the CNS and the periphery. We will then review data that challenge the view that inflammation is solely detrimental, and instead suggest that both suppression and activation of the innate immune system and neuroinflammation may be beneficial, depending on the stage of the disorder. Finally, we will consider several new therapeutic strategies for regulating neuroinflammation, including the immune-modulatory cytokine granulocyte-macrophage colony-stimulating factor (GM-CSF), inhibitors of apolipoprotein E (apoE), and microglial depletion *via* drugs that target the colony-stimulating factor-1 receptor (CSF1R).

### Neuroinflammatory Biomarkers in CSF and Plasma from AD and DS

Studies have found increased cerebrospinal fluid (CSF) levels of immune biomarkers in mild cognitive impairment (MCI) and AD patients (Tarkowski et al., 2003; Galimberti et al., 2006; Jesse et al., 2009; Buchhave et al., 2010; Westin et al., 2012; Kauwe et al., 2014; Counts et al., 2017; Whelan et al., 2019). Peripheral immune cells, such as neutrophils, monocytes, and lymphocytes, also produce (and respond to) inflammatory cytokines, which are significantly upregulated in the blood of AD patients and may also be derived from the CNS (Kim et al., 2008; Diniz et al., 2010; Morgan et al., 2019). Similar increases in blood levels of immune biomarkers are found in people with DS (Petersen and O’Bryant, 2019; Huggard et al., 2020a,b). One inflammation-based hypothesis is that people with DS are in a state of chronic abnormal inflammation, including features of auto-inflammation, across the lifespan that influences all phenotypes and disease risk. Specifically, proteomic analyses of plasma and brain tissue from people with DS revealed dysregulation of inflammatory protein expression, including increases in several pro-inflammatory cytokines, and decreases in numerous complement cascade components (Sullivan et al., 2016, 2017; Zhang et al., 2017). Another study revealed elevated levels of both pro-inflammatory and anti-inflammatory cytokines in plasma from children with DS (Huggard et al., 2020a,b). Activation of astrocytes and microglia, the secretion of inflammatory cytokines (e.g., IL-1, IL-6, and TNFα), and acute phase proteins are observed in both the brains and blood of people with DS, indicating an “inflammatory endophenotype” (Petersen and O’Bryant, 2019). In a cross-sectional analysis of people with DS, plasma glial fibrillary acidic protein (GFAP), a marker of astrogliosis, was found to increase starting in their mid-40s (Hendrix et al., 2021).

### Timing and Effects of Innate Immune System Activation and Neuroinflammation in DS and AD

Epidemiological studies suggest that elevated immune biomarkers in the blood may be evident years prior to the manifestation of clinical symptoms of AD or AD-related dementias (ADRDs) in the typical population (Schmidt et al., 2002; Ridolfi et al., 2013; Leszek et al., 2016; Busse et al., 2017; Wendeln et al., 2018). Higher plasma levels of GFAP are correlated with lower measures of episodic memory and microstructural integrity in AD, MCI, and also in healthy aged donors (Bettcher et al., 2021). As discussed, people with DS also have increasing levels of plasma GFAP starting in their mid-40s (Hendrix et al., 2021). Thus, although initially thought to be a secondary effect of aberrant protein accumulation, changes in the innate immune system and neuroinflammation are now thought to be a core, early feature of both DS and AD that interface with, and may contribute to, clinical manifestations of cognitive disorders and decline (Lucin and Wyss-Coray, 2009; Heneka et al., 2015).

Hsa21 harbors numerous innate immune system and neuroinflammation-associated genes that are therefore triplicated in most people with DS (Table 1). Notably, four genes encoding interferon receptors reside on Hsa21, and interferon-related signaling is upregulated in people with DS (Sullivan et al., 2016; Araya et al., 2019; Powers et al., 2019). The inflammatory response microRNA miR-155 also resides on Hsa21 and is overexpressed in DS (Guo et al., 2019). Proteomic analyses have revealed a striking increase in both pro- and anti-inflammatory cytokines in plasma and brain tissue samples from people of all ages with DS (Sullivan et al., 2017; Zhang et al., 2017; Flores-Aguilar et al., 2020; Huggard et al., 2020b) and in mouse models of DS (Ahmed et al., 2012, 2013; Spellman et al., 2013; Block et al., 2015), which express homologs of Hsa21-encoded inflammation-related genes (Table 1). Together, these findings have led to the hypothesis that DS inherently results in chronic neuroinflammation, including auto-inflammation and astrogliosis (Sullivan et al., 2016; Rachubinski et al., 2019; Snyder et al., 2020). Although non-steroidal anti-inflammatory drugs (NSAIDs) were not therapeutic for AD, it is possible that more selective blockers of the innate immune system might be effective in AD and/or DS, for example, through inhibition of the TLR2-MyD88 interaction or of JAK-1 (Rangasamy et al., 2018; Rachubinski et al., 2019; Tuttle et al., 2020).

**Table 1:** A summary of inflammation-related genes located on HSA21.

Gene	Protein	Function	References	Present in common DS mouse models^#^	AD risk identified in GWAS^@^
*ABCG1*	ATP binding cassette subfamily G member 1	Catalyzes phospholipid and cholesterol efflux and maintains macrophages in an anti-inflammatory state.	Wojcik et al. (2008)	Dp17, Tc1	Wollmer et al. (2007) and Beecham et al. (2014)
*ADAMTS1*	ADAM metallopeptidase with thrombospondin type 1 motif 1.	Secreted protease is known to be induced by IL-1β.	Kuno et al. (1997)	Dp16, Ts65Dn, Tc1	Kunkle et al. (2019), Niu et al. (2019), and Tan et al. (2021)
*ADAMTS5*	ADAM metallopeptidase with thrombospondin type 1 motif 5.	Secreted protease known to be induced by IL-1β and TGFβ.	Yamanishi et al. (2002)	Dp16, Ts65Dn, Tc1	none
*APP*	Amyloid beta precursor protein	Neuronal acute phase protein precursor of Aβ fragments in Alzheimer’s plaques and inducer of IL-1β.	Glenner and Wong (1984), Tanzi et al. (1988), and Barger and Harmon (1997)	Dp16, Ts65Dn, Tc1*	Guyant-Maréchal et al. (2007), Nowotny et al. (2007), and Lv et al. (2008)
*BACE2*	Beta-secretase 2	Cleaves APP for less Aβ and increases IL-1R2, a decoy proteinfor excess IL-1 capture.	Kuhn et al. (2007)	Dp16, Ts65Dn, Tc1	Myllykangas et al. (2005)
*CBS*	Cystathionine beta-synthase	Catalyzes production of hydrogen sulfide bimodalregulation of inflammation.	Sen et al. (2011)	Dp17, Tc1	Beyer et al. (2004)
*CSTB*	Cystatin B	Thiol protease inhibitor involved in Aβ clearance.	Yang et al. (2011) and Maher et al. (2014)	Dp10, Tc1	Kurt et al. (2020)
*CXADR*	CXADR Ig-like cell adhesion molecule	Activation of JNK and p38-MAPK pathways leading toproduction of M1 cytokines.	Yuen et al. (2011)	Dp16, Tc1	none
*DYRK1A*	Dual specificity tyrosine phosphorylation regulated kinase 1A	Serine/threonine and tyrosine kinase that regulates the NFκB pathway and phosphorylates tau.	Latour et al. (2019)	Dp16, Ts65Dn, Tc1	Kimura et al. (2007)
*IFNAR1*	Interferon alpha and beta receptor subunit 1	Activates JAK/STAT mediated anti-inflammatory pathway.	Kim et al. (1997)	Dp16, Ts65Dn	Patel et al. (2021)
*IFNAR2*	Interferon alpha and beta receptor subunit 2	Activates JAK/STAT mediated anti-inflammatory pathway.	Kim et al. (1997) and Boselli et al. (2010)	Dp16, Ts65Dn	none
*IFNGR2*	Interferon gamma receptor 2	Activates JAK/STAT mediated anti-inflammatory pathway.	Boselli et al. (2010)	Dp16, Ts65Dn	none
*PRMT2*	Protein arginine methyltransferase 2	Blocks the actions of NFκB in the nucleus.	Ganesh et al. (2006)	Dp10, Tc1^$^	none
*RCAN1*	Regulator of calcineurin 1	Inhibits calcineurin-dependent transcription and is regulated by STAT2.	Lee et al. (2012)	Dp16, Ts65Dn	Lin et al. (2011)
*RIPK4*	Receptor interacting serine/threonine kinase 4	Necessary for signaling through TNF receptor 1.	Rountree et al. (2010)	Dp16, Ts65Dn, Tc1	none
*RUNX1*	RUNX family transcription factor 1	Transcription factor regulating T-cell function.	Tang et al. (2018)	DP16, Ts65Dn, Tc1^&^	Kimura et al. (2007) and Patel et al. (2011)
*S100B*	S100 calcium binding protein B	Upregulates IL-1β and APP expression, released in responseto TNFα.	Li et al. (1998), Liu et al. (2005), and Donato et al. (2013)	Dp10, Tc1^$^	Lambert et al. (2007)
*SOD1*	Superoxide dismutase 1	Scavenges superoxide radicals producing H_2_O_2_ and O_2_.	Danciger et al. (1986)	Dp16, Ts65Dn, Tc1	none
*TIAM1*	TIAM Rac1 associated GEF 1	Necessary for cytokine-mediated generation of oxidativespecies through NADPH oxidase.	Subasinghe et al. (2011)	Dp16, Ts65Dn, Tc1	none

Some parts of Table 1 are reproduced from Wilcock and Griffin (2013) with permission under the CC BY 2.0 license (http://creativecommons.org/licenses/by/2.0/), Copyright © Wilcock and Griffin (2013); licensee BioMed Central Ltd. Abbreviations: Aβ, β-amyloid; ADAM, a disintegrin and metalloprotease; APP, amyloid beta precursor protein; ATP, adenosine triphosphate; CXADR, coxsackievirus and adenovirus receptor; DS, Down syndrome; IL, interleukin; GEF, guanine nucleotide exchange factor; GWAS, genome-wide associate study; MAPK, mitogen-activated protein kinase; NADPH, Nicotinamide adenine dinucleotide phosphate; RUNX, Runt-related transcription factor; TNF, tumor necrosis factor; TGF, transforming growth factor; JAK/STAT, Janus kinase signal transducer and activator of transcription; JNK, c-Jun N-terminal kinase; TIAM, T-cell lymphoma invasion and metastasis; TNFR, tumor necrosis factor receptor; NFκB, Nuclear factor-kappa B; STAT2, signal transducer and activator of transcription 2. ^#^Tc1 mice express one copy of the human gene and two copies of the homologous mouse gene, while Ts65Dn, Dp10, Dp16, and Dp17 mice express three copies of the homologous mouse gene, as identified in Ahmed et al. (2013). *In Tc1 mice, the *APP* gene is re-arranged, and therefore human APP is not functionally expressed. ^$^In Tc1 mice, the human *PRMT2* and *S100B* genes are duplicated, in addition to two copies of the homologous mouse genes. ^&^In Tc1 mice, the human *RUNX1* gene is partially deleted. ^@^Some relevant GWAS studies were identified using the AlzGene database (Bertram et al., 2007).

### Apolipoprotein E in Inflammation and Neurodegeneration in DS and AD

Inheritance of the *APOE* ε4 allele (*APOE4*) is the strongest risk factor for AD, besides age, with one copy of *APOE4* leading to a three-fold increased risk of AD and two copies leading to a 15-fold increased risk of AD (Corder et al., 1993; Strittmatter et al., 1993; Strittmatter and Roses, 1995). Inheritance of the *APOE4* allele also significantly increases the risk for cognitive decline and dementia in middle-aged people with DS (Rubinsztein et al., 1999; Deb et al., 2000), and it increases the risk of mortality by five-fold compared to non-*APOE4* carriers (Zigman et al., 2005). Furthermore, brain *APOE* expression is significantly upregulated in people with DS compared to the typical population (Lockstone et al., 2007), which might exacerbate DS-associated AD.

Similar to the inflammation/acute-phase protein ACT, apoE is also associated with amyloid deposits (Wisniewski and Frangione, 1992; Wisniewski et al., 1993; Ma et al., 1994; Sanan et al., 1994; Wisniewski et al., 1994). Although not typically considered a neuroinflammatory molecule, *APOE* expression is upregulated by astrocytes and microglia early in the AD pathological process (Keren-Shaul et al., 2017; Krasemann et al., 2017; Kang et al., 2018; Rangaraju et al., 2018), and it also plays a pivotal role in modulating the neuroinflammatory cascade by both Aβ-dependent and Aβ-independent pathways (McGeer et al., 1997; Maezawa et al., 2006; Zhu et al., 2012; Cudaback et al., 2015; Shi et al., 2017; Lin et al., 2018).

**Figure 1:**
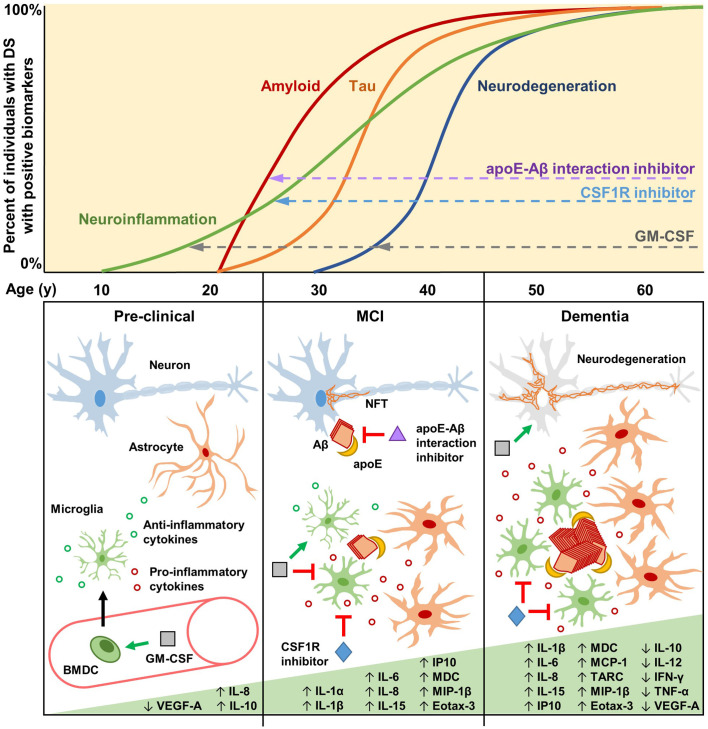
Biomarkers, cytokines, and potential therapeutic mechanisms for neuroinflammation in Down syndrome (DS) and Alzheimer’s disease (AD). Biomarkers of neuroinflammation, amyloid and tau pathology, and neurodegeneration increase over the lifetimes of the vast majority of individuals with DS. Neuroinflammatory biomarkers such as GFAP may begin increasing as early as the late teenage years and progressively increase throughout life (Hendrix et al., 2021). Changes in amyloid biomarkers are detectable after 20 years of age, with *APOE* genotype significantly contributing to risk (Rubinsztein et al., 1999; Deb et al., 2000), likely due to apoE-catalyzed amyloid-β (Aβ) polymerization during the early seeding stages of amyloid formation (Potter and Wisniewski, 2012). Tau pathology is detectable soon after amyloid forms, but it accumulates more slowly. By the age of 40, every individual with DS has the hallmark amyloid and tau neuropathology of AD. Biomarkers of neurodegeneration, such as changes in brain volume and glucose metabolism, are identifiable after 30 years of age, proceeding and generally correlating well with tau pathology. During the pre-clinical stage, GM-CSF may have therapeutic potential by modulating the immune/neuroinflammatory cascade in order to prevent and/or delay amyloidogenesis. GM-CSF stimulates bone marrow-derived cells (BMDCs) to mobilize and extravasate to the brain, or GM-CSF may enter the brain directly, where the resulting activated microglia modulate their cytokine expression and reactivity (Bhattacharya et al., 2015a; Abe et al., 2020). During the mild cognitive impairment (MCI) stage, preceding clinical dementia, Aβ aggregates begin to form, catalyzed by apoE, which then induces intraneuronal tau hyperphosphorylation and neurofibrillary tangle (NFT) formation. Therapeutic molecules that can inhibit the apoE-Aβ interaction (e.g., Sadowski et al., 2004; Pankiewicz et al., 2014; Johnson et al., 2021a) may significantly reduce amyloid deposition, thus preventing/reducing downstream pathologies. Activated microglia and astrocytes, characterized by increased soma size and shortened processes, cluster around amyloid plaques and increase the expression of interleukins and other pro-inflammatory cytokines. At this stage, GM-CSF may act to maintain microglia in a non-activated state, promote anti-inflammatory cytokine expression, and reduce pro-inflammatory cytokine expression (Ahmed et al., 2021; Potter et al., 2021). CSF1R inhibitors may also be beneficial at this stage to reduce the numbers of activated microglia while maintaining quiescent microglia that play important roles in immune surveillance and brain homeostasis (Johnson et al., 2021b). The clinical dementia stage is characterized by neurodegeneration due to widespread NFT formation and chronic neuroinflammation that persists through the mid-40s, 50s, and 60s, during which a majority of individuals with DS have clinical dementia. Some inflammatory markers are reported to decrease during this stage, possibly due to cellular exhaustion and degeneration (Flores-Aguilar et al., 2020). GM-CSF may therapeutically modify neurodegeneration *via* neuroprotective effects, while CSF1R inhibitors may reduce the numbers and effects of chronically activated microglia.

Increasing evidence shows that apoE plays a role in amyloid formation by promoting the aggregation of the Aβ peptide to form insoluble filaments, thereby also inhibiting the clearance of Aβ from the brain (Potter and Wisniewski, 2012; Figure 1). For example, apoE4 catalyzes the formation of neurotoxic Aβ oligomers and fibrils (Ma et al., 1994, 1996; Sanan et al., 1994; Wisniewski et al., 1994; Castano et al., 1995; Golabek et al., 1996; Soto et al., 1996; Manelli et al., 2007; Cerf et al., 2011; Hashimoto et al., 2012; Koffie et al., 2012; Liu et al., 2017). Fortunately, this mechanistic pathway lends itself to therapeutic approaches targeting apoE using antibodies, antisense oligonucleotides, or structural correctors (Brodbeck et al., 2011; Chen et al., 2012; Liao et al., 2014; Huynh et al., 2017; Wang et al., 2018; Xiong et al., 2021), or by targeting the interaction between apoE and Aβ using peptide blockers or small molecule drugs (Ma et al., 1996; Sadowski et al., 2004; Pankiewicz et al., 2014; Johnson et al., 2021a). Recently, high-throughput screens from our lab have identified several Food and Drug Administration (FDA)-approved drugs, including the anti-depressant drug imipramine and the anti-psychotic drug olanzapine, that inhibit the apoE-Aβ interaction and appear to improve cognition in AD patients, and especially in *APOE4* carriers, in our retrospective analyses of human clinical data (Johnson et al., 2021a).

### GM-CSF as a Neuroinflammatory Modulator in AD

Patients with rheumatoid arthritis (RA) have a three- to eight-fold reduced risk of developing AD, suggesting a potential role for inflammation and the innate immune system in AD (McGeer et al., 1996). Although the reduced AD risk in patients with RA was initially attributed to their frequent use of NSAIDs (McGeer et al., 1996), NSAID treatment showed no benefit in clinical trials of either AD or MCI patients (McGeer et al., 2006; ADAPT_FS_Research_Group, 2015).

As an alternative, we hypothesized that intrinsic factors associated with RA pathogenesis itself may underlie its AD protective effect(s). We identified GM-CSF as one such factor that is upregulated in the blood of RA patients and found that subcutaneous injection of GM-CSF for 20 days increased microglial activation, reduced amyloid pathology by more than 50%, and completely reversed the cognitive impairment of transgenic AD mice (Boyd et al., 2010), which has been replicated by other groups (Castellano et al., 2017; Kiyota et al., 2018). Treatment with recombinant human GM-CSF (sargramostim/Leukine^®^) was also associated with improved cognition in cancer patients undergoing hematopoietic stem cell transplantation (Jim et al., 2012). These findings led us to design and carry out a placebo-controlled, randomized, double-blind Phase II clinical trial in mild-to-moderate AD participants, which showed that subcutaneous injection of sargramostim (5 days/week for 3 weeks) was safe, associated with reduced plasma biomarkers of neuronal damage/neurodegeneration (i.e., total tau and UCH-L1), and improved cognition based on Mini-Mental State Examination (MMSE) scores (Potter et al., 2021). These findings suggest that during the early stages of AD, activation of the innate immune system may be beneficial, which has now led to a longer–term trial.

### GM-CSF as an Amyloid-Independent Cognition Enhancer in DS and Normal Aging

People with DS have significant ID throughout their 60-year life expectancy (Bittles et al., 2007), with no treatments available. Several drugs have been tested in mouse models of DS, primarily in Ts65Dn mice, with some promising results showing the rescue of DS-related cognitive deficits. Unfortunately, no such drugs have shown significant benefits in clinical trials of people with DS (reviewed in Gardiner, 2015; Vacca et al., 2019).

Because GM-CSF treatment improved cognition and reduced amyloid in mouse models of AD, we investigated its effects in the Dp16 mouse model of DS. Our findings show that in both Dp16 mice and their wild-type (WT) littermates, GM-CSF treatment improves learning/memory in the radial arm water maze, a hippocampal-based task. In Dp16 mice, GM-CSF treatment also ameliorates the abnormal astrocyte morphology and aggregation and partially normalizes the levels of interneurons (Ahmed et al., 2021). Although GM-CSF treatment evidently improves learning/memory in mouse models of AD by removing amyloid plaques in the brain, it is noteworthy that WT mice and mouse models of DS, including Dp16 mice, do not develop AD amyloid pathology at any age. Therefore, GM-CSF treatment must lead to improved learning/memory in WT and Dp16 mice *via* an amyloid-independent mechanism(s), likely related to its pro-inflammatory or inflammation-modulating activity.

Despite its known pro-inflammatory properties, GM-CSF also affects multiple CNS processes that are consistent with, and provide some insights into, its unexpected beneficial effects on learning/memory in a mouse model of DS. Specifically, GM-CSF promotes recovery from neuronal damage or dysfunction in animal models of stroke (Schneider et al., 2007; Schäbitz et al., 2008; Kong et al., 2009; Theoret et al., 2016), traumatic brain injury (Shultz et al., 2014; Kelso et al., 2015), and acute retinal ganglion cell injury (Schallenberg et al., 2012; Legacy et al., 2013). GM-CSF can cross the blood-brain barrier (McLay et al., 1997) and is also produced within the brain, where numerous cell types express the GM-CSF receptor, including neurons, oligodendrocytes, microglia, astroglia, and endothelial cells, which would allow for both paracrine and autocrine signaling (Baldwin et al., 1993; Sawada et al., 1993).

Many studies have shown that both people with DS and typical aging adults exhibit an auto-inflammatory or “inflammaging” syndrome (Trollor et al., 2012; Frasca and Blomberg, 2016; Ashraf-Ganjouei et al., 2020; Serre-Miranda et al., 2020) that might predict that GM-CSF treatment would be detrimental. However, GM-CSF is not merely a pro-inflammatory molecule. A more accurate description is that GM-CSF modulates the innate immune system, especially in the setting of immune system dysregulation in the periphery and in the brain (Boyd et al., 2010; Bhattacharya et al., 2015a,b; Borriello et al., 2019). Indeed, GM-CSF treatment not only increases the levels of many cellular and cytokine biomarkers of inflammation in the blood of AD patients (e.g., neutrophils, monocytes, lymphocytes, IL-2, IL-6, and TNFα), but also reduces the levels of the inflammatory cytokine IL-8 and increases the levels of the typically anti-inflammatory cytokine IL-10 (Potter et al., 2021). Thus, GM-CSF has a much more complex physiological effect than simply being pro-inflammatory. Furthermore, suppressing the inflammation associated with DS in the periphery may be beneficial in the setting of certain acute disorders (Rachubinski et al., 2019). Thus, growing evidence highlights the complexity of the innate immune system in the context of inflammation in people with DS.

Notably, the fact that we observed improved memory in WT aging mice treated with GM-CSF (Boyd et al., 2010; Ahmed et al., 2021) suggests that GM-CSF has a cognition/memory enhancing activity that is independent of disease and suggests that modulation of the innate immune system may help prevent normal age-related memory decline. In contrast to previous expectations that inhibiting inflammation and the innate immune system would be the most effective therapy for co-morbidities of DS, the beneficial effects of GM-CSF on learning and memory may reflect its stimulating pro-inflammatory activity, its other physiological/cellular effects, or both (Figure 1).

### Therapeutic Modulation of Microglial Numbers and Activation

It is becoming increasingly clear that microglia play multiple, and often disparate, roles at different stages of the innate immune and neuroinflammatory responses in DS and in AD. Accordingly, microglial reduction/depletion has also been investigated as a therapeutic approach to DS, AD, and other neurodegenerative diseases. For example, small molecule drugs targeting CSF1R, which is crucial for microglial proliferation and survival, have been repurposed from cancer indications (Cannarile et al., 2017) and used to modulate microglial levels in the CNS (Figure 1). Indeed, microglial reduction *via* CSF1R inhibition was found to rescue several cognitive deficits in Dp16 mice (Pinto et al., 2020). In AD mouse models, microglial depletion prior to amyloid deposition was shown to be critical for therapeutic efficacy (Sosna et al., 2018; Spangenberg et al., 2019; Son et al., 2020). Likewise, in mouse models of primary tauopathy, characterized by inclusions of the protein tau in neural cells (Kovacs, 2015), CSF1R inhibitors reduced pathological tau aggregation and subsequent neurodegeneration (Mancuso et al., 2019; Shi et al., 2019). However, recent evidence suggests that complete microglial depletion is neither necessary nor desirable for extending lifespan in tauopathy mice and that microglia resilient to CSF1R inhibition exist in a quiescent, non-activated state and may serve important roles in prevention and recovery from tau-induced neurodegeneration (Johnson et al., 2021b). Together, these studies underscore the importance of carefully considering microglial state and function over the disease course in order to appropriately balance microglial stimulation and repression therapeutically.

## Conclusion

Collectively, the recent data indicate that targeted enhancement or inhibition of the innate immune system and inflammatory cytokines can effectively treat DS, AD, and normal aging. These findings provide compelling evidence that the long-held belief that inflammation and innate immune system activation primarily play negative roles in DS, AD, and normal aging must be reassessed. Although GM-CSF is the first modulatory cytokine to exhibit therapeutic potential in inflammation-associated disorders, it serves as a proof-of-principle, and other therapeutic molecules could use a similar approach. Furthermore, combination therapies that pair GM-CSF with small molecule inhibitors of apoE or CSF1R at appropriate neuroinflammatory stages may be particularly effective for treating people with DS and/or AD.

## Author Contributions

All authors contributed to the design and writing of the manuscript. All authors contributed to the article and approved the submitted version.

## Conflict of Interest

HP and TB are two of the inventors on several U.S. patents owned by the University of South Florida, but not licensed. TB is an employee of Partner Therapeutics. The remaining authors declare that the research was conducted in the absence of any commercial or financial relationships that could be construed as a potential conflict of interest.

## Publisher’s Note

All claims expressed in this article are solely those of the authors and do not necessarily represent those of their affiliated organizations, or those of the publisher, the editors and the reviewers. Any product that may be evaluated in this article, or claim that may be made by its manufacturer, is not guaranteed or endorsed by the publisher.
